# Implant Osseointegration Adjacent to a Retained Root Fragment: A Case With 11-Year Follow-up

**DOI:** 10.1155/crid/5547549

**Published:** 2025-07-20

**Authors:** Esra Uyguc, Ekin Yaylaci, Beril Koyuncu, Ovul Kumbuloglu

**Affiliations:** ^1^Department of Prosthodontics, Ege University Faculty of Dentistry, Izmir, Turkey; ^2^Department of Prosthodontics, Altinbas University Faculty of Dentistry, Istanbul, Turkey

**Keywords:** dentin-implant interface, osseointegration, retained root

## Abstract

**Introduction:** There is a growing demand for implant dentistry as the first choice of rehabilitation for treating patients with missing teeth. Clinicians can encounter asymptomatic retained root fragments in edentulous spaces. This case report presents the long-term prognosis of an implant resulting in late failure, with an attached root remnant to the fixture 11 years after implant placement.

**Case Report:** A 68-year-old female patient presented to the clinic with complaints of function related to a maxillary right four-unit implant-supported bridge 11 years after the first insertion of the implants. After the removal of the restoration, a root remnant was found as adhered to the fixture of the middle supporting implant. The implant was placed encroaching upon the mesial part of the residual root fragment left unintentionally, which was discovered accidentally during the bridge removal.

**Conclusion:** Clinicians should show ultimate attention when utilizing the retained root remnants in implant sites, considering the potential risk of hindering osseointegration. However, osseointegration of the encroached implant and root fragment is plausible, with no detrimental short-term impacts observed. A risk–benefit evaluation should be assessed individually, as late failures can still occur.

**Trial Registration:** ClinicalTrials.gov identifier: NCT06671678

**Summary**

• There is a common belief that, in cases of discovering a residual root at a predetermined implant site, surgical removal of the root should be considered because this existing root may lead to infections and hinder osseointegration, resulting in early implant failure.
• This study includes a rarely encountered case showing that osseointegration of the encroached implant with an attached root fragment is plausible, with no observed adverse short-term effects.
• The implants functioned successfully for 11 years but ultimately failed.

## 1. Introduction

In recent years, implant dentistry has been continuously developing, with a gradually growing demand being the first rehabilitation choice that comes to mind in edentulous or dentate patients with missing teeth to restore function and esthetics [1, 2]. Healthy bone and tissues are essential for successful implant therapy [3]. However, early and late complications may occur during this therapy, similar to every medical process [1]. The revised definition of the term “osseointegration,” proposed by Branemark, is the “direct structural and functional connection between ordered living bone and the surface of a load-carrying implant” [4]. For successful osseointegration, direct bone–implant contact is required without interference from the remaining dental or fibrous tissues [5]. Several clinical methods are mentioned in the literature to determine implant stability, such as clinical perception, percussion tests, reverse torque test, Periotest, and resonance frequency analysis (RFA) [6]. Clinical perception is advised to be used during implant insertion [7]. Percussion tests alone are not sufficient since this method is subjectively related to clinician experience [8]. Although radiographic analysis is recommended for preoperative evaluation, it is also commonly preferred for determining implant stability. Distortion in the image, two dimensions, and low resolution are limitations of this method [9]. Being objective but rather expensive, RFA is a noninvasive diagnostic method that measures bone density and implant stability at various moments using vibration with a transducer fixed on the implant [10, 11].

Residual root fragments are the most prevalent radiographic findings in edentulous mouths, whereas they are less frequent in partially dentate mouths [12]. The prevalence of retained roots ranges from 11% to 37% in the population. Careful decision-making regarding their removal should be performed, as they may cause pathology or can be completely symptomless [13]. Under appropriate circumstances, root fragments can remain asymptomatic in situ after a normal healing process consisting of the formation of a cementum layer around the dentin, stimulating bone deposition. Nevertheless, roots fractured during the extraction of nonvital teeth often cause discomfort and pain related to infection [12].

Clinicians occasionally encounter asymptomatic retained root fragments in edentulous spaces [2]. Usually, in cases of discovering a residual root at a predetermined implant site, surgical removal of the root is planned, although this type of extraction is very invasive, expensive, and time-consuming for healing [14]. In such cases, bone augmentation and tissue regeneration are required [15]. Different approaches to rehabilitation including extraction prior to implant placement, dental transplantation, prosthetic and restorative rehabilitation, and computer-guided implant placement bypassing the root fragment have been proposed in such cases when an unerupted/impacted tooth or a retained root fragment is known to be present [16, 17]. During treatment planning, patients demand simple and straightforward solutions with affordable expenses, and clinicians should always consider this fact [15]. The placement of the implant through the residual root fragment or impacted tooth was stated as an alternative method for rehabilitation of such cases by Schwarz et al. making a description of the term “dentointegration” [18]. There is a common belief that retained roots in contact with implants can lead to infections, resulting in peri-implantitis and bone resorption [19, 20]. Some researchers believe that successful osseointegration can also occur even when the implant and the root remnant are in contact [21]. In recent years, there has been a tendency to insert the implants in contact with residual root fragments intentionally [20].

Although the principles of modern implantology have achieved high success rates over the past decades, certain rare clinical situations continue to challenge the conventional treatment protocols. One such scenario is the unintended retention of root fragments in close contact with dental implants, which raises concerns about potential complications, such as impaired osseointegration or late implant failure. Although intentional root fragment preservation techniques—such as the socket-shield approach—have been described in the literature [22], cases involving unintentional retention remain underreported and poorly understood. Therefore, documentation of long-term clinical outcomes in such cases is essential for enriching the evidence base and guiding clinical decision-making when similar situations are encountered.

This case report presents the long-term prognosis of an implant resulting in late failure, with a root remnant on the fixture 11 years after implant placement. All complications and treatments related to the implant site before and after implant placement are discussed in detail.

## 2. Case Report

**Figure 1:**
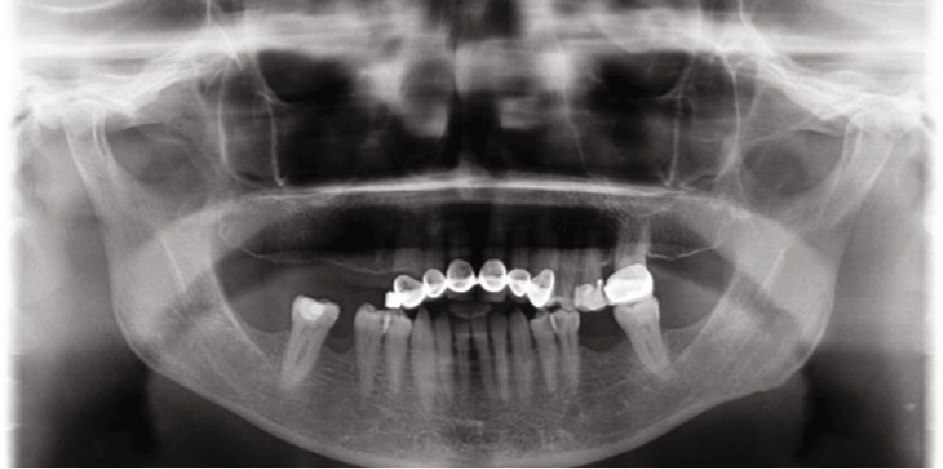
Posterior edentulous areas in the panoramic radiograph.

A 68-year-old female patient presented to Ege University Faculty of Dentistry, Department of Prosthodontics, with complaints of function and esthetics of her upper fixed restorations and removable partial dentures. The patient had no underlying medical condition. After intraoral examination and radiographic evaluation (Figure 1), the removal of the fixed restorations and the evaluation of the prognosis of the abutment teeth were considered, and implant therapy was planned for edentulous sites with adequate mesiodistal distances, except for the right mandible. Following the removal of the anterior fixed restorations, extraction of #13 was planned because it was found to be nonvital with severe cervical caries. The patient was referred to the Oral and Maxillofacial Surgery Department for extraction and implant rehabilitation. In total, four implants (BioHorizons, Birmingham, Alabama, United States) were placed at Positions #13, #14, #16, and #36. The insertion torque values were high, leading to primary stability.

**Figure 2:**
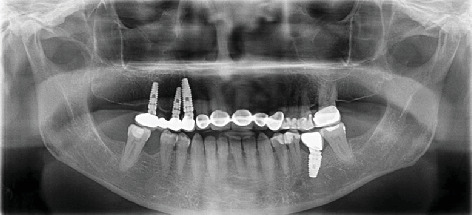
Panoramic radiograph after implant loading.

The implant sites were allowed to heal for 3 months after implant placement. Radiographic analysis and RFA were preferred to determine the secondary implant stability. Gingival formers were placed on the implants as osseointegration proceeded successfully. The maxillary implants were then restored with four-unit cement-retained restorations, and a cement-retained crown was fabricated for #36.  Figure 2 shows the panoramic radiograph after prosthetic rehabilitation was completed.

**Figure 3:**
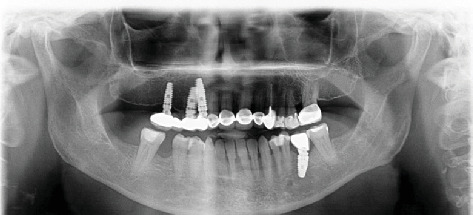
Panoramic radiograph at the 4-year follow-up.

The patient was scheduled for routine follow-up. Four years after implant placement, the patient visited the faculty with an apical abscess and sinus tract related to #23. A successful root canal treatment was performed, and follow-up panoramic radiography was performed during the visit (Figure 3).

**Figure 4:**
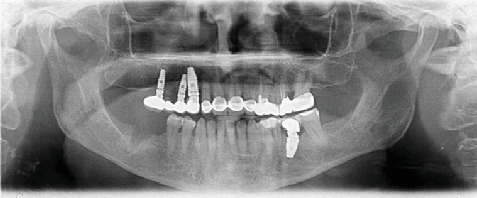
Panoramic radiograph at the 5-year follow-up.

Five years after implant insertion, the patient presented to the clinic with sensitivity to vertical and horizontal percussions on Tooth #25 and tooth fracture on Tooth #47. Treatment involved the extraction of Tooth #47 and root canal therapy of Tooth #25 (Figure 4). Follow-up radiography was then performed.

**Figure 5:**
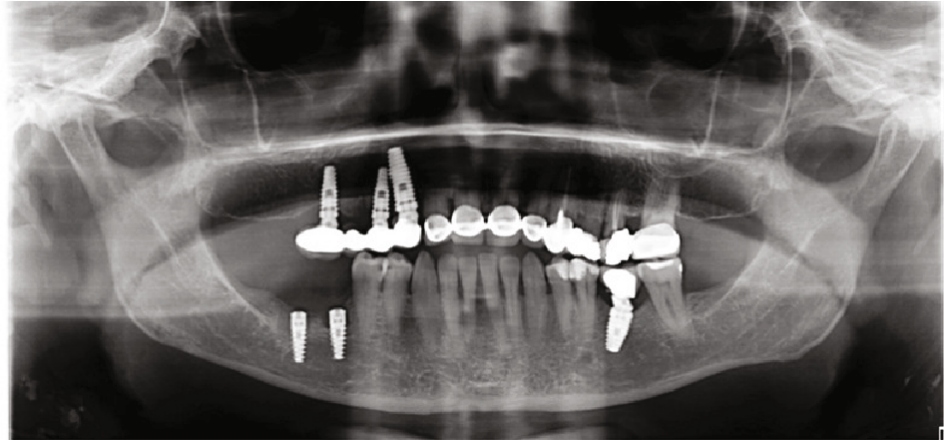
Panoramic radiograph at the 8-year follow-up.

At the 8-year follow-up, the patient visited the clinic requesting implant therapy for the edentulous right posterior mandible. Treatment planning consisted of implant placement at Positions #46 and #47, extraction of Tooth #25 due to severe bone resorption, and renewal of the fixed anterior restorations.  Figure 5 shows the control panoramic radiograph obtained after implant placement in the right posterior mandible. After the removal of the anterior bridge, Tooth #12 was extracted because it was nonvital and had severe caries. Updated treatment planning involved placing two more implants in tooth Positions #12 and #25. All the fixed restorations were successfully completed.

**Figure 6:**
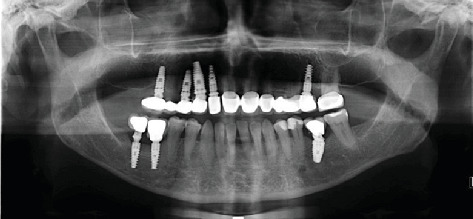
Panoramic radiograph 11 years after implant placement.

Eleven years after the first insertion of the implants in the right maxilla, the patient visited the prosthodontics clinic complaining of mobility in the implant-supported restoration. Prior to the removal of the implant-supported bridge, clinical and radiographic evaluations were performed (Figure 6).

**Figure 7:**
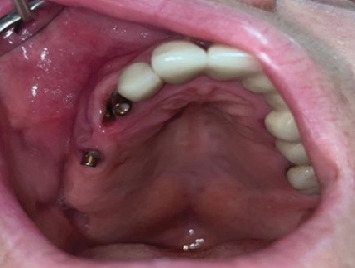
Intraoral image after the removal of the implant-supported restoration.

**Figure 8:**
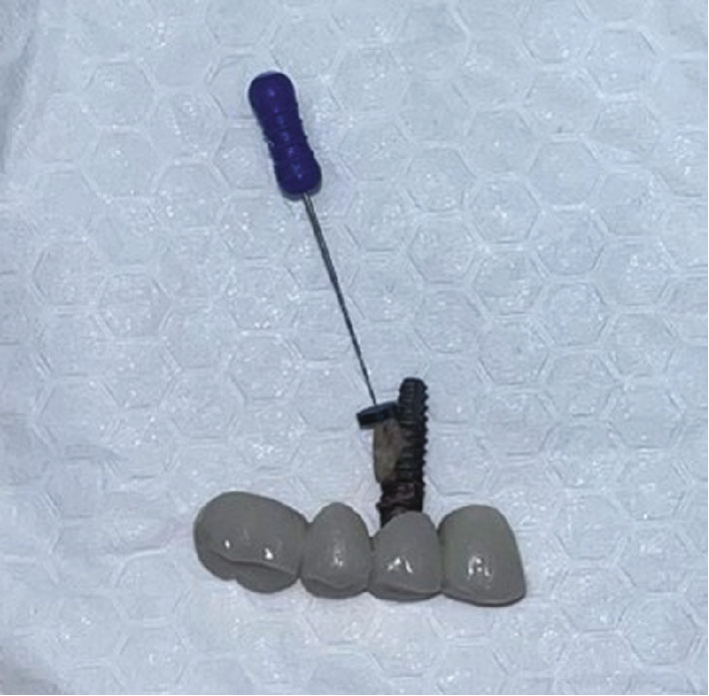
Root remnant retained on the implant fixture.

**Figure 9:**
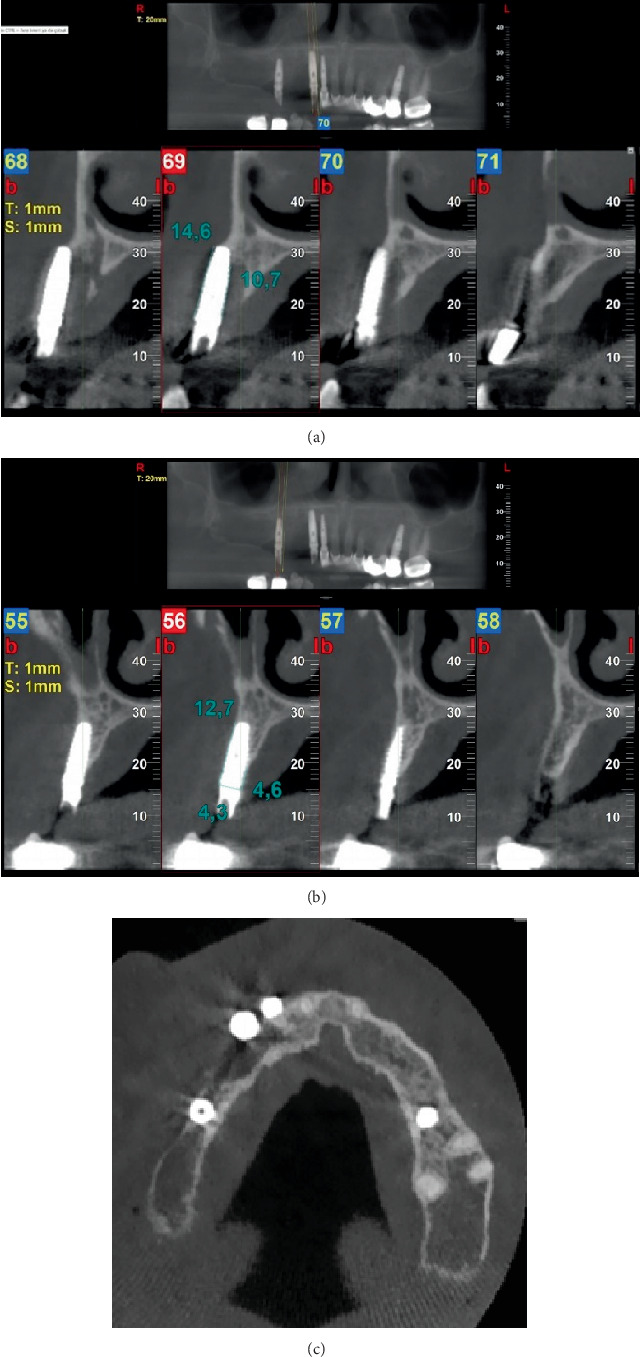
(a) CBCT images of implant #13. (b) CBCT images of implant #15. (c) Axial CBCT image of the related region.

**Figure 10:**
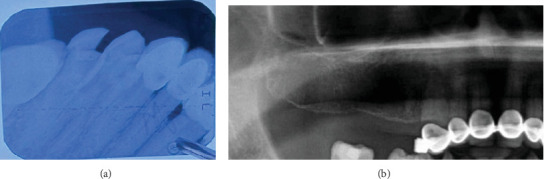
(a) Periapical x-ray before the extraction of #14. (b) X-ray after the extraction of #14.

The restoration was then removed with a failed supporting implant (#14) (Figure 7). After removal, a root remnant was observed to be retained on the fixture of the middle supporting implant (Figure 8). A cone beam computed tomography (CBCT) scan was obtained after removal to analyze the remaining bone density for any other possible implant treatment. According to the CBCT results, there was no cortical bone in the region of the root remnant or implant (Figures 9a, 9b, and 9c). At the time of insertion, implant #14 had been placed encroaching upon the mesial part of the residual root fragment left unintentionally, which was discovered accidentally during removal. Along with the attached root remnant, the removed implant was mailed to the global headquarters of the implant company located in the United States to proceed with further histomorphological analysis to identify any additional findings in terms of osseointegration between the implant and root fragment. Still pending, the results of the analysis are expected to reveal an apposition of cementum-like and/or newly formed bone material at the interface being a testament to strong adherence of the root and fixture. Radiographic images before and after extraction of #14 were retrieved (Figure 10a,b).  Table 1 shows the detailed case summary.

## 3. Discussion

This case report highlights the long-term prognosis of an implant with a residual root fragment attached to the fixture, which resulted in late failure 11 years after placement.

De Smit et al. described the classification of root remnants distinguished by Dachi and Howell in the past. According to this classification, there are two types of root remnants classified considering the susceptibility to the risk of infection: Type 1 (completely embedded in the bone) and Type 2 (fractured due to severe caries exposed in the oral cavity with the potential risk of infection). If there is no denture base or flange close to the Type 1 remnants causing any discomfort, numbness, or nerve compression, these root remnants can remain asymptomatic and may be detected accidentally in radiographic images. Type 2 root remnants commonly account for the development of pathology, with a definitive indication for extraction [13]. These types of root remnants may cause retrograde peri-implantitis, in which the apical portion of the implant is affected while coronal integration continues [20]. In our case, a Type 1 root remnant attached to the implant fixture was accidentally detected after removal. The remnant had no pathology in the last 11-year period enabling the functioning of the implants. However, late failure still occurred, warranting caution.

According to Safi et al., implants interacting with an adjacent root are mostly observed in the posterior maxilla. Researchers have also advised proper treatment planning, including CBCT imaging and surgical guides, to avoid the risk of placing implants in contact with the surrounding tooth [23]. In this case, the relevant implant was placed in the maxillary premolar region, in contact with the unintentionally left root remnant. Only panoramic radiography was performed because the related region was an anatomically risk-free site, and the bone quantity was clinically appropriate. Moreover, at the time of the first insertion, CBCT images were not routinely obtained in all cases.

In their case report, Schwarz et al. stated histological evidence of “dentointegration” between a titanium implant and an unintentionally left vital palatal root of the first molar extracted 8 weeks before. Researchers have stated that vital pulp tissue induces tubular tertiary dentin, which later develops into atubular reparative dentin, preparing the interface for osseointegration. Bone sialoprotein is one of the major components of tubular tertiary dentin, showing a close resemblance to the bone. Dentointegration is established via osteodentin formation. Researchers have provided histological images of the dentointegration interface [18]. In their animal study, Gray and Vernino emphasized that placement of implants unintentionally on root remnants did not cause an inflammatory response [24]. Similar to the study by Nayyar et al. [12], a calcified cementum-like material was observed at the interface of the root fragment and implant fixture in histological analysis [24]. This is likely proof of osseointegration between the root fragments and implants. In this case, a vital root remnant was unintentionally left, but further histological analysis should be performed to examine the histological interface of the implant and the root.

According to a literature review by Perez-Gonzalez et al., the survival rates of implants placed on residual root fragments and encroached through impacted teeth are 76.19% and 97.56%, respectively. The difference in survival rates may be related to long-term intraoral exposure of the remaining roots [2].

Successful osseointegration can be achieved when the implant and retained root are in contact if the root fragment is stable with no pathological symptoms embedded in the bone for years [21]. Szmukler-Moncler et al. presented a case series of patients treated with implants placed through accidentally discovered residual root remnants. All implants in these cases were in place for at least 3 years at that time. The longest follow-up period was 9 years, during which no failures were observed within this period. According to researchers, this technique could be a simplified standard for implant therapy, since the asymptomatic residual roots did not jeopardize implant osseointegration [15]. In their cases, Sohn and Kim preferred to place implants in contact with residual roots in edentulous sites instead of removal. None of the implants placed in the patients showed any pathological findings with clinically stable conditions [14].

In their case series, Davarpanah and Szmukler-Moncler stated that placing implants through ankylosed roots can be considered an alternative approach to the invasive surgical procedure consisting of fragment removal. The researchers emphasized that adverse effects such as fatigue at the osseointegrated part due to any existing periodontal ligament and any periapical/periodontal infection/inflammation endangering implant osseointegration are likely to be observed in these types of cases. All five patients had an average follow-up period of 27 months, with implants functioning successfully and showing no adverse signs [25]. In this case, the implant was placed unintentionally on the residual root and survived for 11 years, with no signs of significant pathology, but ultimately failed.

Having a potential risk of infection at the operative site, unstable root remnants after tooth extraction can presumably hinder osseointegration, resulting in implant failure [26]. Langer et al. emphasized the detrimental effects of implant insertion in contact with undetected root remnants [20]. This situation can result in an apparent decrease in the capacity to withstand occlusal forces [3]. Meticulous curettage is crucial in such circumstances to achieve the complete removal of granulation tissues. Panoramic radiography and CBCT should be performed prior to implant placement [1].

Similar to our case, Nevins et al. presented two cases in which the implants were placed in contact with unintentionally retained root fragments, resulting in late failures after 10 and 4 years of healthy functioning. Initially, the retained root fragments were not noticeable on the pre-evaluation radiography, but as bone resorption progressed, they were easily distinguished [27]. Im and Hong presented a case of implant failure at 3 years postloading, with an osseointegrated root remnant on the fixture. The fragment was left unintentionally and was not detected preoperatively. Historical analysis revealed hypercementosis on the root, a cementum-like matrix, and newly formed bone, providing osseointegration [3].

Guarnieri et al. reported a case of early implant failure (1st year of implant placement) due to the root remnant left unintentionally at the surgical site. The remnant was found to be strongly adhered to the fixture [28]. Similarly, Lee et al. reported a case of early implant failure related to a hard tissue remnant consisting of a thick cementum layer. Two implants were placed at Positions #36 and #37 after the teeth (#37 and #38) were extracted because of the severe cavities. A two-stage surgical protocol was preferred because of the poor primary stability of implant #37. Three months later, osseointegration failure was suspected, and panoramic radiography revealed a radio-opaque remnant unintentionally left around the related implant [1]. The hard tissue consisting of cementum jeopardized the early osseointegration process in this case, resulting in the removal of the failed implant with the surrounding tissues and placement of new implants after the bone-healing period. The reason for this failure is thought to be the remnant of an infected tooth with severe caries. In our case, late implant failure occurred, with the root remnant strongly adhered to the fixture.

If the remaining root fragment had been detected prior to implant placement, alternative treatment strategies could have been considered. These may have included guided bone regeneration (GBR) after the extraction of the root or placing implants using a bypass approach. Various augmentation techniques, such as alveolar distraction, bone grafting, and GBR, have been widely utilized to reconstruct resorbed alveolar bone and facilitate proper osseointegration and long-term implant stability under functional loading. Among these, GBR is one of the most frequently employed methods for managing peri-implant bone deficiencies and restoring alveolar bone volume [29]. In cases of large adjacent edentulous areas, slight tilting of the implant to eliminate the encroaching upon the residual root can be considered as well [15]. However, there are contradictory ideas regarding the remaining root fragments in scientific literature. Another technique is the “socket-shield technique,” in which the buccal part of the root is left intentionally with minimum mobility before the implant placement with a success rate of 96% [30]. This technique preserves bone and soft tissue volume, which is very significant for providing esthetics [27]. The most important advantage of this technique is sectioning the root intentionally with no mobilization. However, in cases of retained root fragments, there is a risk of the root remnant being mobilized during tooth extraction, which may lead to bacterial contamination. Keeping the root remnant nonmobilized is the key factor for the high success rate of the socket-shield technique [22].

## 4. Conclusion

Removal of the root fragment should be carefully considered because of the disadvantages of invasive surgery. Clinicians should show ultimate attention when utilizing the retained root remnants in implant sites considering the potential risk of hindering osseointegration. In the present case, implant loss occurred in the region where the contact between the implant and root remnants was observed. This may suggest either mechanical retention or potential biological integration; however, owing to the lack of histological evidence, a definitive conclusion cannot be drawn. Yet no other complications were documented during the follow-up period, though long-term viability may still be compromised. Although the implant remained functional in the oral cavity for a considerable period, it ultimately failed resulting in an unsuccessful outcome. A risk–benefit evaluation should be assessed individually, as late failures may occur. Further in vivo studies including more cases with longer follow-up periods are required to verify the long-term prognosis of implants placed through the residual roots. The clinical takeaways are as follows:

• The potential for long-term asymptomatic function of dental implants in contact with retained root fragments has been demonstrated in select cases.
• Clinical decision-making should be individualized, taking into account patient-specific anatomical, systemic, and risk-related factors.
• Meticulous preoperative imaging and comprehensive treatment planning are imperative for the detection of residual root fragments and formulation of an evidence-based surgical approach.

## Figures and Tables

**Table 1:** Detailed case summary table.

**Time/year**	**Clinical findings**	**Procedures performed**	**Involved teeth**	**Figure**
Initial (Year 0)	Intraoral and radiographic evaluation performed	Removal of fixed restorations; prognosis of abutment teeth assessed; implant therapy planned for edentulous areas (excluding right mandible)	—	Figure 1
Initial	#13 found nonvital with severe cervical caries	Extraction of tooth #13	#13	—
Initial	Implant placement	Four implants placed	#13, #14, #16, #36	—
Postrestoration	Restoration of implants	Four-unit cement-retained prosthesis in maxilla; cement-retained crown in mandible	#13, #14, #16, #36	Figure 2
4th year	Apical abscess and sinus tract observed	Root canal treatment performed; follow-up panoramic radiograph taken	#23	Figure 3
5th year	Sensitivity to percussion on #25, tooth fracture on #47	Extraction of #47, root canal therapy on #25	#25, #47	Figure 4
8th year	Patient requested implant therapy in right posterior mandible	Implant planning for #46 and #47; extraction of #25 due to bone loss; anterior restorations planned for renewal	#25, #46, #47	Figure 5
8th year	Anterior bridge removed; #12 found nonvital with severe caries	Extraction of #12; implant planning for #12 and #25	#12, #25	—
11th year	Complaint of mobility in implant-supported prosthesis	Restoration removed due to failure of supporting implant	#14	Figures 6 and 7
11th year	Unexpected finding during removal	Root remnant observed to be retained on the middle supporting implant	—	Figure 8
11th year	CBCT evaluation	No cortical bone observed in the area of the root remnant and implant	—	Figures 9a, 9b, and 9c
11th year	Radiographic images before and after the extraction of #14	Radiographic images retrieved	#14	Figure 10a,b

## Data Availability

The data that support the findings of this study are available from the corresponding author upon reasonable request.
